# *Hieracium maccoshiana*, a new Scottish hawkweed related to *H. dovrense* (Section Alpestria, Asteraceae)

**DOI:** 10.3897/phytokeys.3.920

**Published:** 2011-05-30

**Authors:** Timothy C. G. Rich

**Affiliations:** Department of Biodiversity & Systematic Biology, National Museum Wales, Cardiff CF10 3NP, UK

**Keywords:** Asteraceae, *dovrense*, Endangered, *Hieracium*, lectotype, *maccoshiana*, new species, Norway, Scotland, Section, *Alpestria*

## Abstract

A new species of hawkweed *Hieracium maccoshiana* T.C.G.Rich **sp. nov.** is described. It is related to the Norwegian *Hieracium dovrense* Fr., but differs in the shape and toothing of the stem leaves and in having glabrous-tipped ligules. It is endemic to the county of Sutherland, Scotland where it has been recorded from four sites. A lectotype of *Hieracium dovrense* is designated.

## Introduction

In 1897, E. S. Marshall and W. A. Shoolbred collected a hawkweed from rocks on the north side of Ben Loyal, Sutherland, Scotland, which they attributed to the Norwegian species, *Hieracium dovrense* Fr. (Marshall and Shoolbred 1898). This identification has been followed in all subsequent accounts of *Hieracium* in Britain (Linton 1905; Pugsley 1948; Sell and West 1965, 1968; Sell and Murrell 2006). Four more sites were discovered in Sutherland at Creag na h-Uidhe, Foinaven and Rhiconich (two sites) in the 1960s and 1970s by A. G. Kenneth and A. McG. Stirling, and the plants were again attributed to *Hieracium dovrense*.

*Hieracium dovrense* Fr. was described by E. M. Fries in 1848 from several alpine localities in Norway, particularly in the Dovre area (Fries 1848). Fries stated that he had seen Scottish specimens of ‘*Hieracium amplexicaule*’ (presumably in its broad sense), which looked like his *Hieracium dovrense*, but the material he saw has not been traced.

In 2005, D. McCosh suggested that I should visit one of the Rhiconich sites to see if *Hieracium dovrense* was still present, and further visits were carried out in 2010 as part of a revision of *Hieracium* section *Alpestria* (Fr.) Arv.-Touv. in Britain. During this work I compared the Sutherland plants against a specimen in BM cited in Sell and West (1965) as a ‘provisional lectotype’ and other material. I came to the conclusion that they were not conspecific, and differed consistently in leaf shape and toothing and in hairiness of the ligule tips. The Sutherland plants also do not match the descriptions of any of the other subspecies of Zahn’s (1921) general species *Hieracium dovrense* or other Scandinavian material I have seen.

## Methods

Material from Sutherland and of Norwegian *Hieracium dovrense* was studied in the field in 2010 (see Supplementary file 1; *Hieracium dovrense* survey 2010; vouchers in NMW) and in the following herbaria in detail: MANCH, NMW, S and UPS. In addition, material from BM, CGE and E was also consulted. A description was drawn up following the format of Sell and Murrell (2006) for comparison with other British species.

The number of leaves on the stem is an important character in section *Alpestria*, but different taxonomists do not adopt a consistent method of counting leaves. The problem arises as leaves on the stem with inflorescences in their axils can also be termed bracts, which may or may not be counted. Furthermore, although there is a gradual transition from stems leaves to bracts, the smallest bracts at the top of the main stem may differ markedly in shape from the lower leaves and may be included or excluded. Here, all leaves and inflorescence bracts on the main stem are counted.

## Taxonomic treatment

### Lectotype of Hieracium dovrense Fr.

Sell and West (1965: 93) discussed the complicated typification of *Hieracium dovrense* in detail. They provisionally designated a lectotype from material in BM labelled ‘Norveg. centr. a Dovre in Finmarkiarn copiose, M. N. Blytt’, which was sent out as *Hieracium cydonaefolium* Vill. in Fries’s Set 11 no. 12 but noted that the specimen was badly damaged and that a fresh designation should be made if better quality original material were found (NOTE: Under Article 7.11 of the ICBN (McNeill et al. 2006) typifications only have priority if “definitely accepted as such by the typifying author”; usage of the word ‘provisional’ in this context means that the typification of Sell and Murrell (1965: 93) can be superseded). Courtesy of Thomas Karlsson, an undamaged specimen from this set has now been found in S (accession number S09.31085, http://andor.nrm.se/kryptos/fbo/kryptobase/large/S09-031001/S09-31085.jpg) and is hereby designated as the lectotype of *Hieracium dovrense* Fr.

#### Hieracium maccoshiana

T.C.G.Rich sp. nov.

urn:lsid:ipni.org:names:77111568-1

http://species-id.net/wiki/Hieracium_maccoshiana

Figs 1 2 D–N 

##### Latin

Rosula basalia sub anthesi fere absenti. Foliis caulinis 4–8 ellipticis dentibus magnis. Pedunculis pilis multis stellatis pilis eglandulosis simplicibus multis pilisque glandulosis paucis. Bracteis involucralibus 2–2.5 mm latitudinis pilis stellatis paucis pilis eglandulosis simplicibus multis pilisque glandulosis multis. Ligulis apice glabris. Stylis obscuris.

##### Holotype:

**Scotland**. Sutherland: by small stream, Allt na Cuile, Rhiconich, 58.4470, -4.9300, 150 m alt., 26 July 2010, M. Jannink (**NMW**, accession number V.2010.1.213).

**Figure 1.**
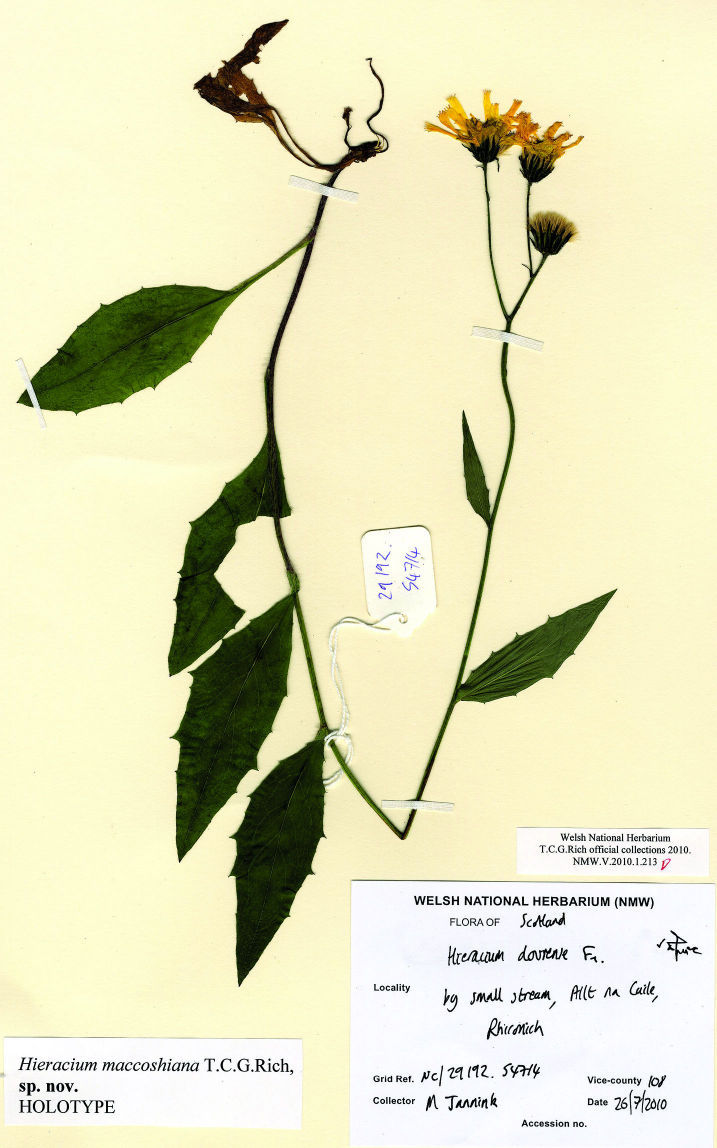
Holotype of *Hieracium maccoshiana* (NMW).

##### Description.

Stem to 50 cm, pale green, slightly purple–tinted below, slender to fairly robust; stellate hairs sparse and mainly above; simple eglandular hairs long, pale, sparse and glabrescent to dense (especially below); glandular hairs very small, occasional; sparsely and minutely puberulous. Basal leaves absent or withering before or up to flowering time or rarely persistent, few. Lamina elliptical or subrotund; apex obtuse–mucronulate; base attenuate; margins remotely denticulate to coarsely dentate; with a few, pale, medium simple eglandular hairs above and below or nearly glabrous above. Petioles winged and broadened at base, with numerous medium to long, pale simple eglandular hairs. Stem leaves and inflorescence bracts (3–)4–8, 2–10 × 0.8–3.5 cm, gradually decreasing in size upwards, pale green on upper surface, paler beneath. Lamina of the lower leaves elliptical; apex obtuse–mucronulate to acute; base attenuate; margins denticulate to sharply and irregularly dentate with ascending teeth; petiole winged, semiamplexicaul. Lamina of the median leaves elliptical; apex obtuse–mucronulate to acute; base rounded or abruptly contracted, sessile, semiamplexicaul; margins denticulate to sharply and irregularly dentate with large or small, ascending, mammiform teeth. Lamina of the upper leaves lanceolate; apex acute to acuminate; base cuneate, sessile, semiamplexicaul, margins denticulate to shallowly dentate. All stem leaves with stellate hairs few to sparse on both surfaces; simple eglandular hairs few to numerous below and nearly glabrous above, pale, medium. Inflorescence usually with 2–9(–12) capitula, rather compactly cymose. Peduncles 1–5 cm (acladium 0.3–2 cm), suberect, slender; stellate hairs sparse to dense; simple eglandular hairs few to numerous, short to medium, dark–based; glandular hairs few, very short, black. Capitula 20–30(–45) mm in diameter, subtruncate at base. Involucral bracts 9–11 × (1.7–)2.0–2.5 mm (the outermost from *c.* 5 mm long), all linear–lanceolate, blackish–green; apex obtuse; stellate hairs sparse, often with a tuft at the apex; simple eglandular hairs many, short to medium, dark–based; glandular hairs many, very short, black. Ligules yellow, glabrous–tipped. Styles discoloured. Achenes 4.0–4.5 mm, blackish–brown. Margins of receptacle pits long–dentate. Chromosome number 2n = 36 (cf. Sell and Murrell 2006, as *Hieracium dovrense*).

**Figure 2.**
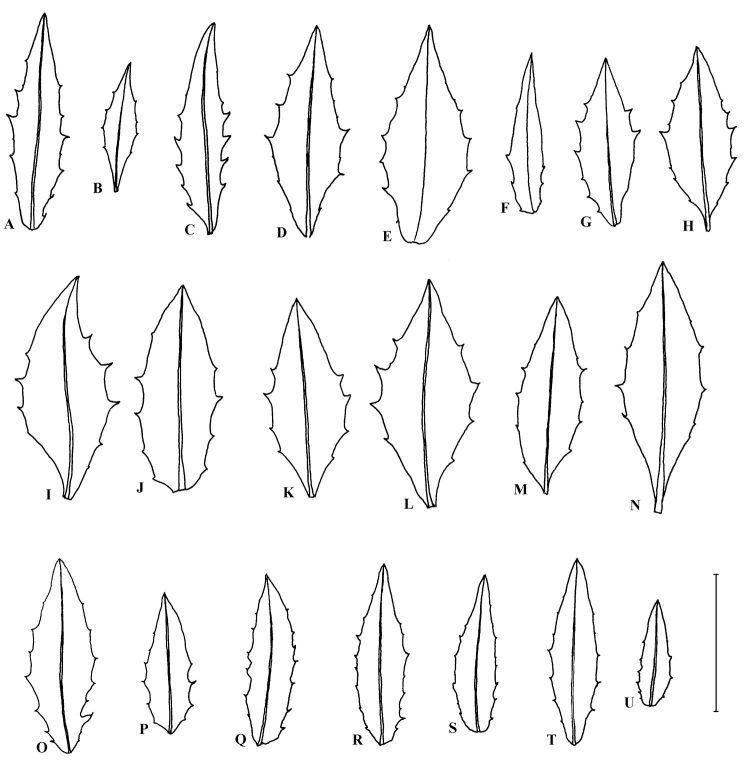
Middle stem leaves. **A–C** possible *Hieracium maccoshiana*. **A–B** Ben Loyal (NMW 28.131.5185). **C** Ben Loyal (BM). **D–N** *Hieracium maccoshiana*. **D–F** Rhiconich gorge (NMW V.2005.1.159). **G–H** Foinaven (CGE). **I–L** Rhiconich (CGE). **M–N** Craig na H’Uidhe (CGE). **O–U** *Hieracium dovrense*, Drivstua, Dovrefj, Norway (S). **O–Q** S09-16213. **R** S09-16217. **S** S09-16224. **T–U** S09-16222. Scale bar 5 cm.

##### Distribution.

**Figure 3.**
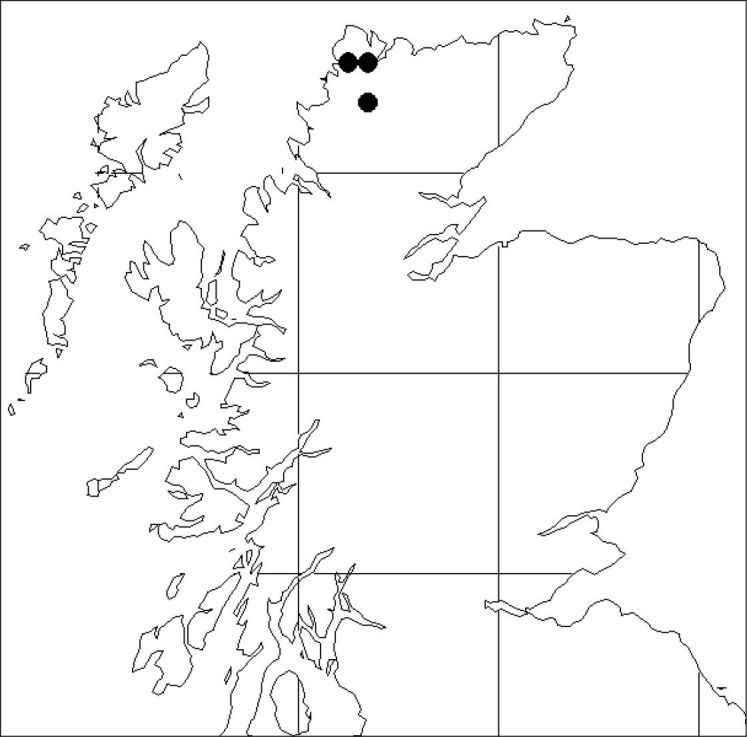
Distribution of *Hieracium maccoshiana* in Scotland.

Endemic to Sutherland, Scotland, where it is known from four sites in Sutherland: in a gorge and on a burn side near Rhiconich; on a burn side in Coire Dùail, Foinaven; and on rocks at Creag na h-Uidhe (Fig. 3). The status of plants from Ben Loyal remains to be clarified when it has been refound in the field.

##### Ecology.

Mountain cliff ledges and rocks, in rocky ravines and on riversides. It mostly occurs out of the reach of large herbivores such as deer or sheep as, like most leafy hawkweeds, is likely to be selectively eaten. The altitudes range from 130–414 m. It flowers from late July to August, and is probably apomictic. Some plants may produce sparse pollen and others none (Thomas et al. 2011).

**Etymology**. The epithet honours David J. McCosh for his work in mapping British and Irish *Hieracium* taxa over the last 30 years, and for mentoring me. The spelling ‘*maccoshiana*’ follows recommendation 60C.5.a of the International Code of Botanical Nomenclature (Vienna Code McNeil et al. 2006).

**Conservation status**. Surveys in 2010 revealed about 60 plants in four sites (see supplementary file 1: *Hieracium dovrense* survey 2010). It is thus best considered at IUCN (2001) threat status ‘Endangered’ due to the small population size.

## Discussion

*Hieracium maccoshiana* is characterized by the absence of a basal rosette at flowering, the relatively few (4–8), elliptical stem leaves with large teeth, the relatively hairy peduncles, the relatively broad (2–2.5 mm) involucral bracts with few stellate hairs but many simple and glandular hairs, the glabrous ligule tips and the discoloured styles.

*Hieracium maccoshiana* is quite variable in hairiness and in size, with very small plants growing on dry, exposed rocks, and larger plants in sheltered situations. The basal rosettes have usually withered by flowering time but may persist when sheltered in tall vegetation. Material from Rhiconich cultivated by J. R. N. Mills became much more robust with more stem leaves with larger teeth, and developed many branches down the stem with more capitula (MANCH).

Plants from Ben Loyal tend to have slightly narrower, elliptical leaves (Fig. 2, A–C) and look more similar to Norwegian *Hieracium dovrense* than plants from the other populations but have glabrous ligule tips. I have provisionally grouped them with *Hieracium maccoshiana* but have been unable to refind them in the field yet to carry out more detailed studies.

No British species is closely allied to *Hieracium maccoshiana*. Four other section *Alpestria* species occur in mainland Scotland and England. *Hieracium perthense* F.N.Williams (*Hieracium carpathicum* auct. angl.) and *Hieracium dewari* Syme have many more (6–20), broader, more hairy stem leaves, narrower (1.1–2.0 mm) involucral bracts with numerous glandular hairs, and hairy tips to the ligules. *Hieracium mirandum* P.D.Sell and C.West has ovate to lanceolate stem leaves with more densely hairy peduncles, and narrower (1.5–2.0 mm), more sparsely hairy involucral bracts. *Hieracium solum* P.D.Sell and C.West has a basal rosette at flowering, a few, nearly entire stem leaves and nearly glabrous peduncles. Sixteen section *Alpestria* species occur in Shetland, of which *Hieracium australius* (Beeby) Pugsley is most similar to *Hieracium maccoshiana* but has generally more (5–12) stem leaves which are more hairy on the upper surface, has larger (to *c.* 35 mm) capitula, and has less hairy peduncles without glandular hairs.

*Hieracium maccoshiana* differs from *Hieracium dovrense* having elliptical middle stem leaves, typically (14–)25–40 mm wide and 2.0–2.8 times as long as wide, with few, large teeth (Fig. 2), and glabrous ligule tips. *Hieracium dovrense* has elliptical to narrowly elliptical middle stem leaves 13–25 mm wide and 2.6–3.4 times as long as wide with more, smaller teeth (Fig. 2) and ciliate ligule tips. Sell and West (1965) described the achenes of Scandinavian plants as 2.0–2.5 mm long and dark brown but I suspect these were immature as fruits of both species I have seen are very similar.

Unfortunately, when Fries described *Hieracium dovrense*,he cited the earlier name *Hieracium cydonae*[*ii*]*folium* Vill. as a synonym thus invalidating the name. As Stace (1998) designated *Hieracium dovrense* as the type species of section *Alpestria*, a separate proposal will be required to conserve the name *Hieracium dovrense* Fr.

## Conclusion

*Hieracium maccoshiana* is a new endemic species from Scotland. It is a member of *Hieracium* section *Alpestria* and differs from *Hieracium dovrense* within which it has previously been included by the shape and toothing of the stem leaves and in having glabrous ligule tips. The status of plants from Ben Loyal remains to be clarified.

## Supplementary Material

XML Treatment for Hieracium maccoshiana
